# A Rare Cause of Fever of Unknown Origin: Amoebic Liver Abscess

**DOI:** 10.1590/0037-8682-0577-2022

**Published:** 2023-02-20

**Authors:** Serdar Aslan, Tümay Bekci, Ramazan Orkun Önder

**Affiliations:** 1 Giresun University, Faculty of Medicine, Department of Radiology, Giresun, Turkey.

**FIGURE 1:**
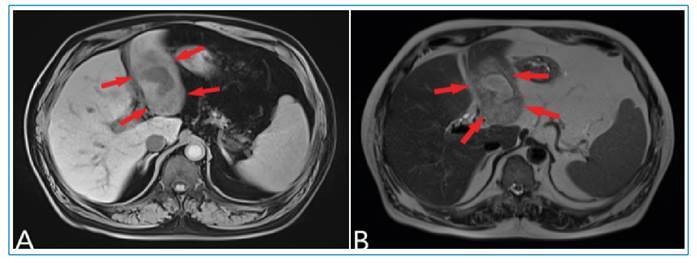
**(A)** Hypointense lesion detected on T1-weighted images extending inferiorly at the second segment of the liver (arrows); **(B)** Hyperintense lesion on T2-weighted images (arrows).

**FIGURE 2:**
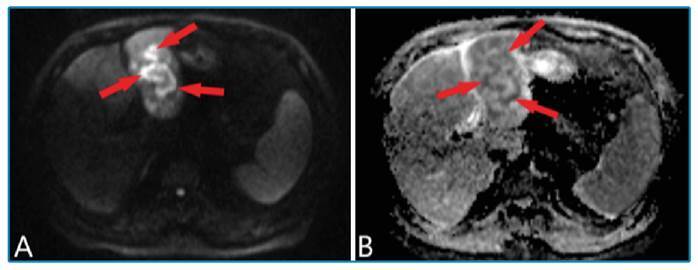
**(A, B)** Diffusion-weighted images and apparent diffusion coefficient (ADC) map reveals restricted diffusion within the lesion (arrows).

**FIGURE 3:**
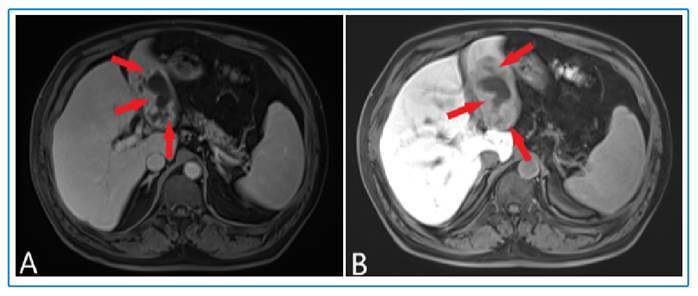
(A) Gadolinium enhancement except for the cystic components (arrows); (B) In the hepatobiliary phase, gadoxetic acid material is retained less than in the liver parenchyma (arrows).

A 68-year-old man presented to our emergency department with complaints of malaise and fever. His medical history was unremarkable, except for arterial hypertension and tap water use. His body temperature was 37.9 °C, and physical examination revealed tenderness in the upper right quadrant upon palpation. Laboratory tests showed elevated levels of C-reactive protein (CRP) (212 mg/L), white blood cells (14.3 × 10^9^/L), and erythrocyte sedimentation rate (ESR) (88 mm/h). The patient was admitted with an initial diagnosis of a fever of unknown origin. Ultrasonography revealed a solitary hypoechoic lesion with cystic components in the left lobe. Contrast-enhanced magnetic resonance imaging revealed an appearance consistent with an abscess with peripheral contrast enhancement, hypointensity on T1-weighted images, and hyperintensity on T2-weighted images extending downward in the second segment of the liver. Additionally, perilesional edema was observed (Figures 1,2,3). Percutaneous liver abscess drainage was performed under general anesthesia. *Entamoeba histolytica* antibody seropositivity was detected. The diagnosis of amoebic liver abscess (ALA) was confirmed. The patient was treated with metronidazole (500 mg) thrice daily for 14 days. *E. histolytica* is a pseudopod-forming protozoan parasite that causes proteolysis and tissue lysis. Humans are the natural hosts. Amoebic infection occurs by the ingestion of mature cysts via feces-contaminated food, water, or hands. The most common extraintestinal manifestation is ALA. Liver abscess develops in< 4% of patients^1^ ^-^ ^3^. ALA should be considered in patients with fever of unknown origin, especially in those with upper right quadrant sensitivity, elevated CRP and ESR, and eosinophilia.
